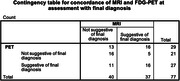# The use of MRI, FDG‐PET and CSF biomarkers in young‐onset cognitive disorders: real‐world clinical evidence on the concordance of investigations

**DOI:** 10.1002/alz.092359

**Published:** 2025-01-09

**Authors:** Molly J Whitmore, Christopher G S Gilmartin, Christopher Tench, Elizabeta Mukaetova‐Ladinska, Akram A. Hosseini

**Affiliations:** ^1^ School of Medicine, University of Nottingham, Nottingham, Nottinghamshire UK; ^2^ Mental Health and Clinical Neurosciences Academic Unit, School of Medicine, University of Nottingham, Nottingham, Nottinghamshire UK; ^3^ Clinical Neurology, Nottingham University Hospitals NHS Trust, Nottingham, Nottinghamshire UK; ^4^ NIHR Nottingham Biomedical Research Centre, Nottingham, Nottinghamshire UK; ^5^ School of Psychology, University of Leicester, Leicester, Leicestershire UK; ^6^ Academic Neurology, Nottingham University Hospitals NHS Trust, Nottingham, Nottinghamshire UK

## Abstract

**Background:**

Cognitive disorders are clinical diagnoses informed by history, cognitive testing and investigations including MRI, FDG‐PET and CSF biomarkers. These investigations do not always correlate however. This study sought to investigate the concordance between MRI, FDG‐PET and CSF biomarkers with final diagnosis in a real‐world clinical context.

**Method:**

This is a prospective longitudinal cohort study (CogNID, NCT03861884, IRAS 250525) of a referral cognitive neurology NHS service (n=345). MRI and FDG‐PET at assessment were appraised for whether they were suggestive of the final diagnosis. An interim analysis was performed on a quality assured dataset of those aged ≥40 and <70 years old.

**Result:**

Cohort characteristics: 40≤age<70 (n=265), MRI results available (n=245), FDG‐PET available (n=129), CSF analysis available (n=104). Interim results (n=77): Kappa coefficient of concordance of 0.15 (95% CI 0.03‐0.28) between MRI and FDG‐PET for suggestion of final diagnosis, demonstrating a poor level of agreement. MRI at time of assessment was suggestive of final diagnosis in 48% of cases and FDG‐PET in 56%. The positive predictive value for a final diagnosis of Alzheimer’s Disease based on CSF analysis in keeping with Alzheimer’s continuum was 64%, with a negative predictive value of 75%.

**Conclusion:**

Investigations must be viewed holistically in combination with a clinical history and cognitive examinations. Initial results at time of assessment are often not indicative of the final diagnosis, and may be discordant. Follow‐up and repeat investigations where appropriate are critical for improving diagnostic accuracy.

*CG&MW are joint first authors